# Mending the gap: Measurement needs to address policy implementation through a health equity lens

**DOI:** 10.1093/tbm/ibae004

**Published:** 2024-02-25

**Authors:** Gabriella M McLoughlin, Shiriki Kumanyika, Yanfang Su, Ross C Brownson, Jennifer O Fisher, Karen M Emmons

**Affiliations:** Department of Social and Behavioral Sciences, Temple University College of Public Health, Philadelphia, PA, USA; Implementation Science Center for Cancer Control, Brown School, Washington University in St. Louis, St. Louis, MO, USA; Department of Community Health and Prevention, Drexel University Dornsife School of Public Health, Philadelphia, PA, USA; Department of Global Health, School of Public Health, University of Washington, Seattle, WA, USA; Implementation Science Center for Cancer Control, Brown School, Washington University in St. Louis, St. Louis, MO, USA; Division of Public Health Sciences and Siteman Cancer Center, Washington University School of Medicine, St. Louis, MO, USA; Department of Social and Behavioral Sciences, Temple University College of Public Health, Philadelphia, PA, USA; Department of Social and Behavioral Sciences, Harvard T. H. Chan School of Public Health, Boston, MA, USA

**Keywords:** policy implementation, implementation science, health equity, measurement

## Abstract

Policies represent a key opportunity to improve the health outcomes of populations, and if implemented well, can reduce disparities affecting marginalized populations. Many policies are only evaluated on whether they elicit their intended health outcome. However, a lack of understanding regarding if and how they are implemented may hinder the intended impact overall and on addressing health disparities. Implementation science offers an array of frameworks and methodological approaches for assessing policy delivery, yet few examples exist that meaningfully include health equity as a core focus. This commentary describes the importance of equity-informed implementation measurement by providing case examples and implications for assessment. In addition, we highlight examples of emerging work in policy implementation grounded in health equity with suggested steps for moving the field forward. The ultimate goal is to move toward open-access measurement approaches that can be adapted to study implementation of a variety of policies at different stages of implementation, driven by input from marginalized populations and implementation practitioners, to move the needle on addressing health disparities.

## Teaser

How can policy implementation science meaningfully integrate health equity to drive implementation measurement?

**Implications**

**Practice:** Although many policies exist to improve health outcomes affecting marginalized populations, very few efforts have been made to measure implementation, limiting understanding of whether these policies are successful.

**Policy:** Embracing health equity as a major component in policies and their implementation could enhance their impact on population health, increasing likelihood of success.

**Research:** By meaningfully integrating health equity to inform policy implementation measurement, researchers can better assess if and how these policies work to improve health of marginalized populations.

## Introduction

Disparities in the prevalence of chronic diseases such as overweight/obesity, cancer, type II diabetes mellitus, and other noncommunicable diseases have worsened in recent years, particularly since the onset of the COVID-19 pandemic [1–4]. These disparities affect populations identified as a minoritized racial or ethnic group and those living in low-income circumstances, who are disproportionately at risk for a wide range of preventable diseases, due to systemic racism and exclusion. Accordingly, efforts to address these disparities include enactment of policies designed to improve inequitable community or institutional contexts for behaviors such as healthy eating and physical activity. To enhance clarity, we include our operational definition of health equity, developed by Braveman [5]: “Health equity means that everyone has a fair and just opportunity to be as healthy as possible. This requires removing obstacles to health such as poverty, discrimination, and their consequences, including powerlessness and lack of access to good jobs with fair pay, quality education and housing, safe environments, and health care. For the purposes of measurement, health equity means reducing and ultimately eliminating disparities in health and its determinants that adversely affect excluded or marginalized groups.” For the purposes of health equity in relation to any given policy or initiative, the disparity outcome measured is context specific. The impact of the policy is reflected in its potential to create or mitigate differences in access or benefits affecting the community or population of interest [6]. We wish to make this clear and avoid generalized statements about health equity as the goal, which may weaken the intended benefits of policies.

Policies adopted at the national, state, and local levels have the potential to heavily reduce health disparities. Unfortunately, to date policies that are designed to improve health equity are often poorly evaluated and their implementation determinants poorly understood [7], limiting our ability to evaluate their impact and/or learn from the implementation process. Systematic reviews conducted to understand the status of policy implementation measurement [8, 9] have emphasized a lack of tools grounded in health equity constructs. For this commentary, we operationalize equity-informed policy implementation measurement as, “*Efforts to understand, through a variety of qualitative and quantitative data sources, whether, why, and how a policy or initiative was implemented to account for social and economic circumstances that may unfairly limit policy effectiveness in certain population groups.*” To achieve equity, marginalized groups need to receive additional support to have health outcomes that are not limited by social and economic circumstances [10]. Racial equity in policy development and implementation is a major priority of the US Federal Government, further highlighting the significance of such targeted efforts [11, 12].

Implementation science can fill an important gap between policy and practice [7, 13, 14], by illuminating the gray area between policy and impact through clear, rigorous methodology. However, although the notion of policy implementation has been studied for over 30 years [15], the lack of attention paid toward *measuring* implementation presents a clear gap in the field, and limits our understanding of how and why certain policies might be effective or ineffective, and where the gaps remain [7, 13]. Furthermore, there is a clear need for more attention to measurement of policy implementation to understand content, determinants, processes, and outcomes of policies that promote health [12, 16–18]. This would facilitate understanding of how and why policies do or do not work and greatly inform our ability to ensure that policies have a positive and equitable impact on health.

Equity-focused policy implementation has challenges and opportunities. In a review of a range of policy approaches and their role on health disparity reduction [19], most policies were shown to either reduce health inequalities or were neutral toward inequalities, yet some appear to increase inequalities (e.g. low emission zones in cities). One systematic review showed that low emission zones improved overall air quality and health outcomes, but the health benefits were greater for the wealthiest residents [20]. Wealthier areas might benefit more from improved air quality and reduced traffic, while less affluent areas might not share these benefits. Healthcare access might also moderate the differential impact of air quality on health as the more deprived populations have less access to healthcare after the exposure to air pollutants. Furthermore, settings or communities experiencing inequities may lack the resources to conduct evaluation and measurement. In some cases, policymakers may lack the political will to assess the impacts of inequities. These gaps highlight opportunities, which we will discuss.

Accordingly, the purpose of this commentary is to (i) explain the implications of measurement gaps and consequences for implementation of policies and (ii) examine the status of existing equity-informed policy implementation measurement tools and highlight gaps that need to be filled. We provide recommendations for researchers working in policy implementation and health equity, to address key measurement gaps and advance the field.

## An Example: Implications of Measurement Gaps in School Nutrition Policy During COVID-19

At the onset of the COVID-19 pandemic, in 2020, all public schools across the USA were forced to pause in-person instruction and thus one of the largest food safety net programs—school breakfast and lunch provision—changed drastically in its implementation. Rates of food insecurity increased dramatically following the declaration of a national emergency and closures of schools and businesses [21, 22]. These closures meant lack of access to universal free school meals for high-poverty families. Universal free school meals provide breakfast and lunch at no charge to all students as a result of the Community Eligibility Provision of the United States Department of Agriculture Healthy Hunger Free Kids Act which targets schools serving high-poverty families to mitigate food and nutrition insecurity [23–25].

National- and local-level studies revealed significant gaps that limited the extent to which those school meal service policies effectively reached students in need [22, 26]. Specifically, only 39% of states/territories provided comprehensive implementation guidance to schools and districts [26], with many jurisdictions only providing information about open meal pickup sites but no other information such as how to best implement the new waivers, promote meal participation, or other best practices. As a result, implementation varied across states and jurisdictions due to lack of communication to implementers on the ground, limiting their impact on addressing disparities in food insecurity [26]. Significant barriers to understanding the determinants or effectiveness of these implementation strategies were that (i) measurement efforts were happening in rapid-cycle fashion and (ii) there was no alignment of methods and measurement tools for school meal implementation. Thus, a key issue is a lack of measurement surrounding policy implementation, limiting our understanding of downstream implementation determinants, processes, and outcomes. This lack of information undermines the effect of policies designed to mitigate disparities, as we cannot tell if/how they have been effective and thus inequity remains. Below we use this example to demonstrate the importance of equity-informed measurement of implementation in reducing disparities and promote population health.

## Our Charge: Highlighting Equity-Informed Measurement as a Priority in Implementation Science

Although policy surveillance systems are generally well developed [27, 28], specific attention is needed on equity-focused surveillance data from multiple sectors outside of healthcare (e.g. education, economic development, and criminal justice). Several public health systems have begun to develop these policy-relevant indicators and metrics [29, 30], including most recently the World Health Organization [31], but more work is needed to integrate policy-relevant data with *rigorous implementation measurement*. Given this gap, we are faced with the challenge of improving ways to assess policy implementation with a primary focus on mitigating disparities. Without such attention to implementation measurement, we risk further harm to marginalized populations by not understanding the unintended consequences of “implementation as usual.”

**Figure 1:**
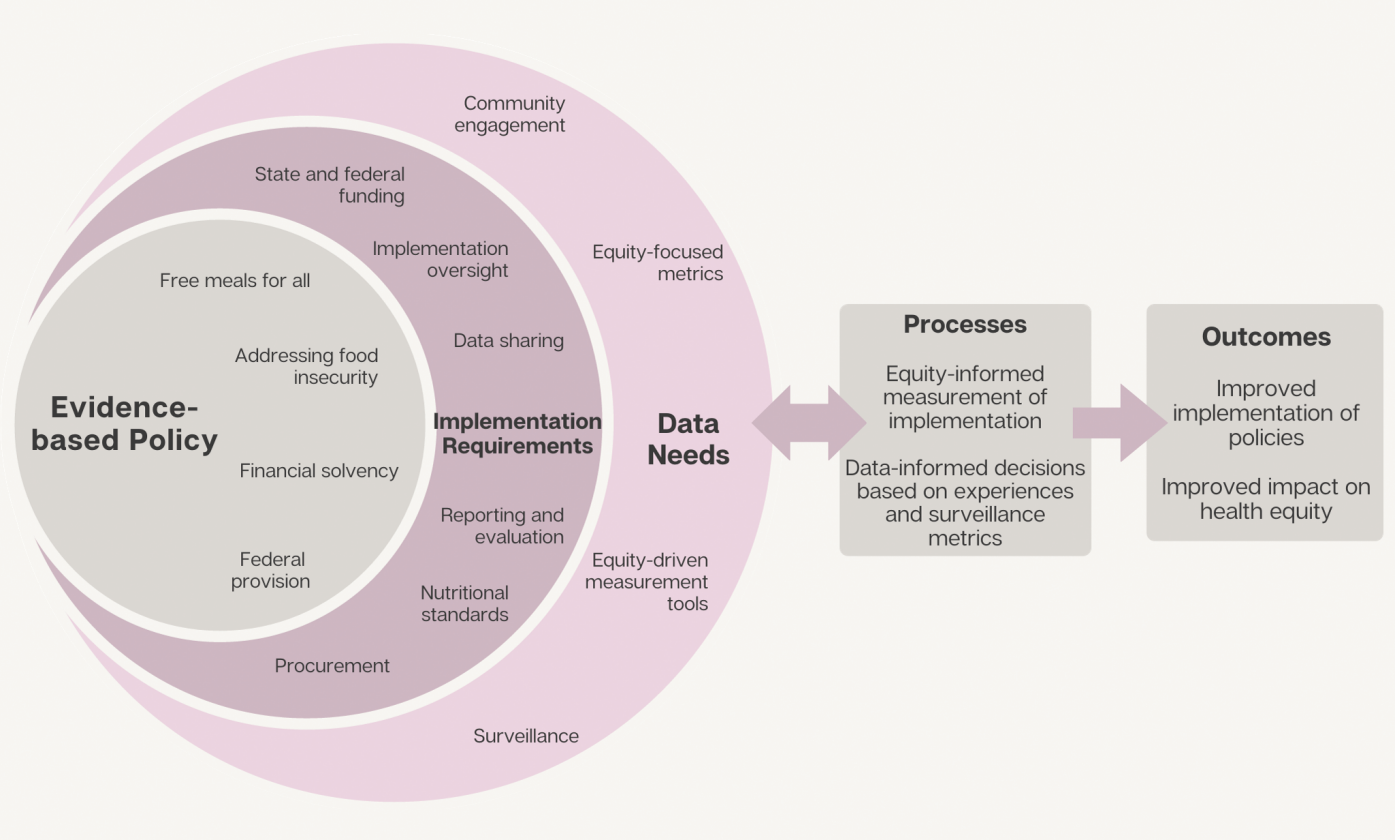
Conceptual framework showing the potential impact of equity-informed policy implementations measurement. Impact of equity-informed measurement

Below we provide a conceptual framework to outline the processes of policy implementation measurement grounded in health equity, integrating the example described above with the Community Eligibility Provision (Fig. 1). Moving from left to right, the first segment shows the Evidence-Based Policy being adopted and the key components of that provision. The second segment outlines the establishment of the requirements of policy implementation (i.e. Implementation Requirements), which must be understood prior to designing implementation measurement. In the context of Community Eligibility, conditions set at the federal and state level dictate how the policy should be implemented, as there are specific nutritional standards to meet and procedures for reimbursement. As with all policies, there is often implementation oversight and enforcement from different levels of jurisdiction and those tasked with implementation provide data on policy compliance. As with Community Eligibility, school food service staff must document how many students receive school meals, the nutritional quality of these meals, and the cost of procurement, among other metrics, through a rigorous data sharing process.

The layer surrounding the policy implementation requirements shows the data needs that should be considered in understanding implementation determinants, processes, and outcomes and at multiple levels of implementation, given the issues with a “top-down” approach previously described in the Community Eligibility example. Through engaging partners and potential policy beneficiaries/end users in each of these levels, using equity-informed tools and existing data, the goal is that ongoing refinements can be made to implementation over time. This could ensure that schools and districts have the implementation support needed and the knowledge of how school meals are being served, where the implementation gaps are, and challenges which may be impacting low-resource schools or those serving minoritized groups more than others.

There is also a reciprocal and dynamic relationship between the policy, its requirements, the data needs, and the implementation process, indicated with a bidirectional arrow linking the process component to the ecological component. As such, the processes may change in response to the implementation measures and data coming from both those tasked with implementation (i.e. practitioners) in addition to the metrics and surveillance data that public health agencies and other entities collect. For example, during the school closures when schools pivoted to providing free school meals for all, it would have been extremely beneficial to have open and continuous information sharing among practitioners, policymakers, and researchers so that practice-informed policy changes could be made in real time. The final section illustrates the intended outcomes of this process, if executed effectively; ultimately, we propose this will lead to a better understanding of if/how a policy elicits impact and to what degree this equitably benefits those most marginalized in society.

## Tools and Frameworks Exist to Guide Equity-Informed Policy Implementation Measurement

**Figure 2:**
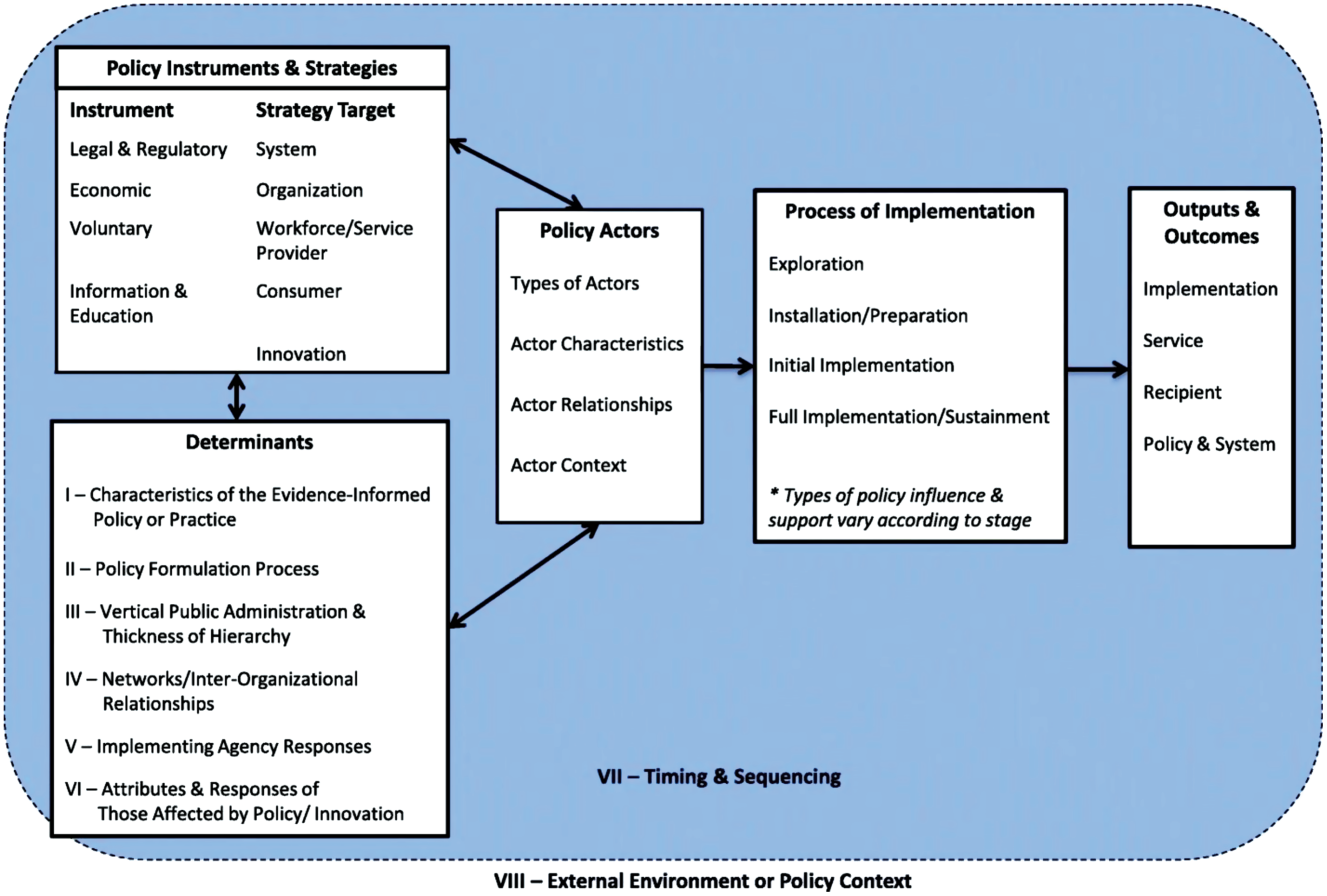
Policy process and determinants framework [35]. Policy implementation framework

Many frameworks have been developed for use in implementation research [32], and there is a substantial body of frameworks for health equity [6, 33, 34]. It is not a goal of this commentary to develop a new framework. Instead, we seek to highlight examples of existing frameworks coupled with measurement approaches to assess existing resources for researchers, policymakers, and other practitioners involved in policy implementation and evaluation. One salient framework is the Policy Process and Determinants framework [35] by Bullock *et al.* to enhance understanding of how aspects of the policy continuum can influence its overall impact (see Fig. 2).

The framework contains both policy implementation process and determinant components to acknowledge that these require equal attention in evaluation. The model also synergizes with two widely used implementation science frameworks: the Exploration, Preparation, Implementation, Sustainment (EPIS) framework [36] and the Implementation Outcomes framework [37]. The integration of these frameworks emphasizes that policies need to be implemented effectively once “adopted” by a certain level of jurisdiction. A primary focus is on understanding the key determinants driving policy implementation, the interaction between determinants and actors, and how these dynamic forces shape the processes and ultimately outcomes of policy implementation. These components are often ignored in policy research, which impedes understanding of *whether*, *how*, and *why* a certain policy elicits its intended outcome.

To address the second goal of this paper, we conducted a search of both peer reviewed and gray literature to find examples of equity-informed policy implementation measurement tools. This was not systematic, since similar reviews had already been conducted and not found any implementation measurement tools grounded in health equity [8, 16]. To be included in this commentary, frameworks needed to outline *specific measurement approaches* that would lead to a better understanding of policy implementation; they needed to also highlight what specific health disparities/marginalized groups were to be affected and a clear plan for how measurement would guide their approach. Below we describe three equity-informed policy implementation evaluation initiatives from a variety of settings, their facets/constructs, and the linkage to the Bullock framework to understand coverage of evaluation and identify gaps in measurement.

## The Racial Equity and Policy Framework

The Racial Equity and Policy (REAP) framework [38] was developed by Dr Jamila Michener to address disparities in the way policies, specifically Medicaid, are developed and implemented with three central tenets of Disproportionality, Decentralization, and Voice. The first: Disproportionality, denotes the problem when policies unfairly benefit or burden certain racial groups over others. Considerations for equity-informed policy implementation include assessing how people and communities of color are included in policy networks, how they are represented in the different levels of policymaking procedures, and how they are constructed or depicted in policy ideas. These issues are an essential part of mitigating disproportionality by ensuring that those most marginalized have a voice in the policymaking procedures.

Decentralization pertains to a needed shift away from top-down approaches whereby policies are made at the federal level with little autonomy for state and/or local levels in its implementation. Key questions that one should use to measure decentralization include: “Are key institutions located at the national, state, and/or local level?” and “What are the economic and political contexts within which policy is being enacted and implemented?” The central premise is that such distribution of decision-making power will enhance equity of a policy and ultimately its implementation. Voice addresses the ability of marginalized communities to contribute to policymaking and the environment. By incorporating such voices and perspectives, key items to measure include whether policymakers are “giving meaningful voice to people of color and those most affected” and the “role of communities of color in shaping these contexts.”

The REAP framework has been promoted within the medical literature, and serves as an important guide for measurement, however no measures have since been developed to facilitate evaluation of policy implementation through a health equity lens. Despite this drawback, we did feel that the REAP framework deserved inclusion in this paper because these four key constructs could easily be used to ground development of interview guides in qualitative phenomenological research to understand implementation of policies from an implementer and end-user perspective. In the article itself, an example of how REAP was used to understand implementation of Medicaid work requirements highlighted key questions that could be asked for each construct of the framework. For example, the construct of Voice could guide questions such as “Who is being included in the decisions to implement this Medicaid policy change?” and “Are state and federal decisionmakers substantively engaging tribal communities?” which could easily be used as a starting point for an interview guide. This would facilitate better understanding of how/why certain populations are not being reached by the medical community within a certain healthcare system or municipality and enhance data sharing practices to build trust among communities most marginalized from medical care.

## The Policy Equity Analysis Tool

The Policy Equity Analysis Tool (PEAT) [39] was developed at the Great Lakes Equity Center to address equity in education policy. The tool serves as an evaluation framework and comprises six key areas: Legal (policy meets legal mandates), Research Base (derived from evidence of effective outcomes for all students), Responsive to Context (district needs, marginalized populations), Efficient (use of time and resources), Educative (informs stakeholders of purpose), and Accountable (specifies responsibilities). The tool allows educators and education leadership to engage in a plan-do-study-act [40] exercise which may bring them closer to implementing policies that disrupt inequitable education practices and outcomes for marginalized youth.

Since its inception in 2015, this tool has been integrated in many school districts across the USA [41], and similar versions of planning tools have been adopted among larger urban districts and [42] state-level boards of education [43]. The tool itself is intended for districts to evaluate themselves and includes Likert-scale questions and free-response questions to facilitate self-reflection. This is a starting point for measurement development; advances to the PEAT could include (i) testing of psychometric properties of these questions, (ii) gaining community partner and school community member (i.e. students and caregivers) feedback on this tool to enhance external validity, or (iii) a rigorous case study, triangulating data from the PEAT to secondary and/or qualitative data within a given school district.

## Measure4Change

Measure4Change [44] is a large ongoing project and comprehensive support service developed by equity experts at the Urban Institute. This project emerged in response to a critical need around policy evaluation identified in a World Bank meeting in 2013 and has grown into a large project designed to build capacity in nonprofit settings for equitable policy implementation. Within this project is a Performance Measurement Playbook that gives community and/or nonprofit organizations “plays” to help support measurement and evaluation of equity in every aspect of their work. The Data Collection section of this playbook provides guidance for how organizations can utilize qualitative and quantitative data, in addition to existing metrics, to help evaluate equitable implementation. This is more process-focused, guiding the “how” of measurement, as opposed to the other two examples which provide more content regarding “what” to measure.

**Figure 3:**
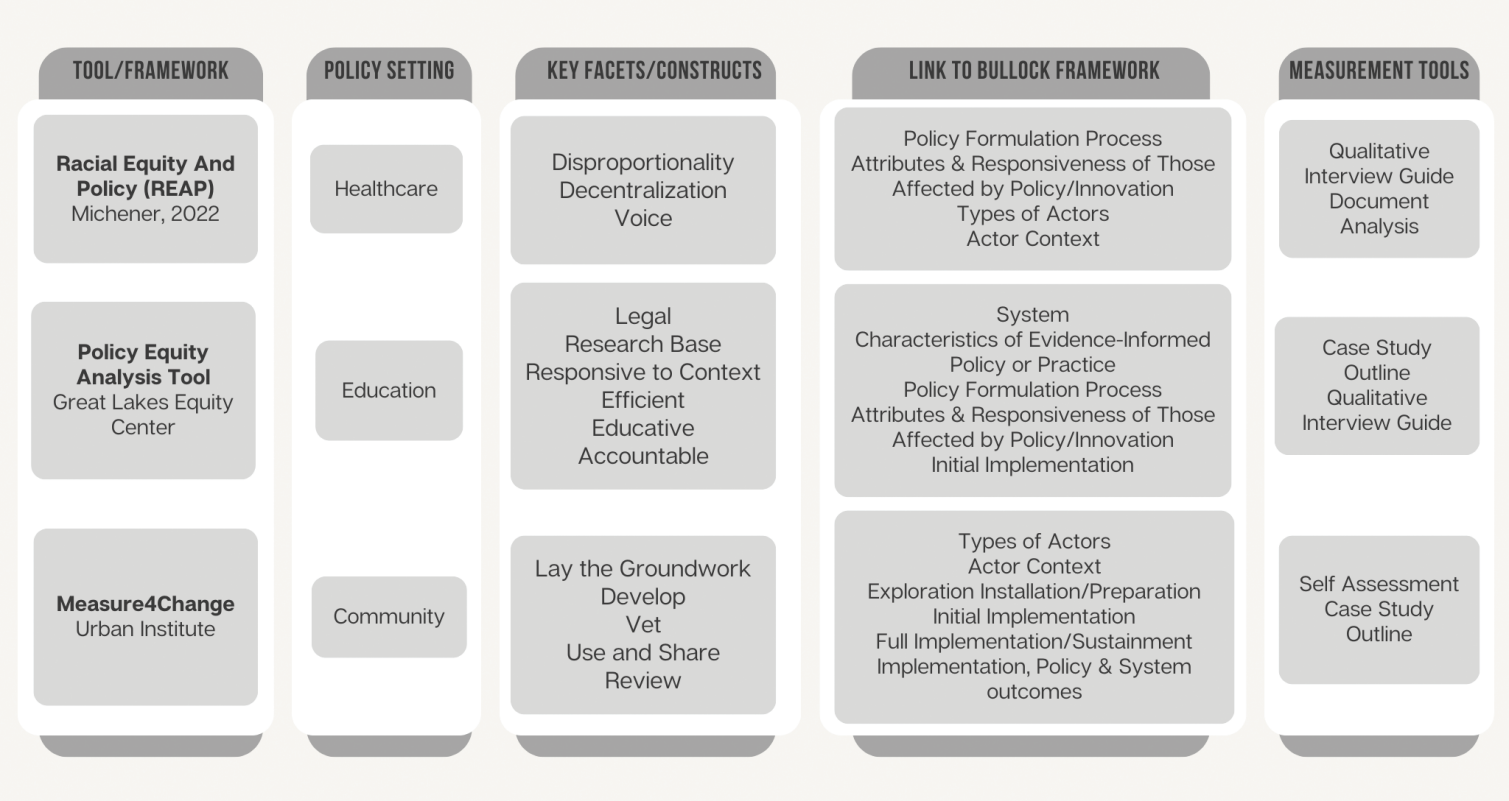
Existing health equity tools and frameworks aligned to Bullock framework. Health equity tools and measurement opportunities

Guidance is presented around several different areas including data collection, data management, data analysis and communication, and racial equity approaches. Currently, the Measure4Change project partners to train nonprofit organizations in equitable implementation. Although the Measure4Change playbook provides the steps for organizations to develop their measurement procedures and evaluation, it does not provide examples of data collection tools or other assessment documents. This may be an intentional decision so as not to appear overly prescriptive regarding how organizations should plan their evaluation efforts. Fig. 3 provides a visual overview of all three measurement resources and how they align to the Bullock framework with suggested starting points for measurement tools, to facilitate utilization in different public health settings.

## How Do We “Mend the Gap” in Equity-Informed Policy Implementation Measurement? Recommendations for Future Directions and Research

Although much work has been conducted to bridge the research to practice gap, we still lack policy-related implementation knowledge because of inadequate or inappropriate measurement tools. We therefore need to move beyond the notion that policy adoption or compliance is the end goal and think deeply about how we engage end users and policy providers in the implementation process. Below we offer three key recommendations for advancing policy implementation measurement tools, grounded in health equity.

1) *Build on the work of others*: The use of conceptual frameworks helps underpin the main components of policy implementation, and some important tools and frameworks have been developed with a focus on pragmatic implementation support [38, 39, 44]. However, gaps remain such as meaningfully integrating health equity and social justice frameworks with implementation science [45]. One example of such integration is a measurement development study which seeks to iteratively develop measurement tools for school policy implementation [13]. This is a two-part study in which the first aim is to collaboratively select constructs from health equity and implementation science frameworks, ranked by school-based practitioners, researchers, and others involved with school policy implementation. The second aim is to establish face and content validity of measurement tools (i.e. surveys and interview questions) with a variety of participant groups (i.e. students, caregivers/parents, teachers, administration, and food service). The end goal of this project is a set of equity-informed implementation measurement tools for policies, initially developed for the school setting. An adaptation guide will then be developed to help researchers and practitioners use these tools for a variety of policies in the chronic disease prevention setting, facilitating evaluation of policy implementation.
2) *Prioritize lived experience*: Often the experiences of those “on the ground” are ignored when evaluating implementation of a policy. The study described above prioritizes voices of those with lived experience and implementation expertise (i.e. teachers, food service, parents/caregivers, students, etc.) to develop policy implementation measurement tools through qualitative research (i.e. cognitive interviewing) to establish face and content validity [13]. For the field of implementation science to also prioritize these perspectives, a paradigm shift is essential, in addition to a larger focus on qualitative research to ensure that the voices of those in marginalized populations are truly heard. In the current, dominant paradigm, strict reliance on internal and/or criterion validity unfortunately serves as a hindrance to advancing how we understand and conceptualize health equity. If time/funding is limited, researchers should at least refer to secondary data and/or existing literature showing perspectives of marginalized groups and end users to guide selection of constructs and questions to include in policy implementation measures.
3) *Design for dissemination*: Researchers should collaborate to develop a shared measures repository—this would reduce time/effort in reinventing the wheel, provide open-access tools for continuous usage, and enhance surveillance of policy implementation. As such, disseminating beyond the peer-reviewed publication and sharing measurement tools with nonprofit organizations, medical providers, state/local organizations, and departments of public health demonstrates a commitment to knowledge translation and improving capacity of under-resourced entities to improve their understanding of policy implementation. One resource of such design for dissemination is a new web tool that guides researchers through their own dissemination process, ensuring that they are taking multiple steps to engage different audiences and enhance the translation of research to practice [46].

Equity-focused dissemination of research findings could include several core elements. At a systems level, funders should provide incentives for researchers to engage in meaningful ways with audiences experiencing disparities (e.g. through requirements for dissemination and supplemental funding). To improve dissemination processes, researchers should engage with community partners (i.e., those faced with implementation and those directly affected by the policy) early and often in the research process [47]. Products for dissemination could be improved by refining messages that resonate with key populations and developing communication materials in collaboration with the audience of focus that reflect the images, narratives, and outcomes of interest to populations experiencing disparities.

## Conclusion

This commentary highlights the need for equity-informed, policy-specific implementation measurement grounded in the needs and voices of those tasked with implementation and recipients of such policies. We identify existing resources and recommend steps to advance the field. Policy implementation science studies have received significantly less attention and extramural funding than those focused only on intervention outcomes in healthcare and nonclinical settings yet offers unmatched potential for addressing health disparities and population health [7, 48]. Thus, policy-specific implementation measurement must deal with general policy execution but with provisions for meaningfully addressing equity issues. Building on the work of health equity and social justice researchers to develop meaningful and pragmatic open access implementation measurement tools is likely to drive the field of policy implementation forward.

## Welfare of Animals

This article does not contain any studies with animals performed by any of the authors.
